# Imatinib inhibits SARS-CoV-2 infection by an off-target-mechanism

**DOI:** 10.1038/s41598-022-09664-1

**Published:** 2022-04-06

**Authors:** Romano Strobelt, Julia Adler, Nir Paran, Yfat Yahalom-Ronen, Sharon Melamed, Boaz Politi, Ziv Shulman, Dominik Schmiedel, Yosef Shaul

**Affiliations:** 1 Department of Molecular Genetics, Weizmann Institute of Science, Rehovot, Israel; 2 Department of Infectious Diseases, Israel Institute for Biological Research, Ness Ziona, Israel; 3 Department of Immunology, Weizmann Institute of Science, Rehovot, Israel; 4 Present Address: Fraunhofer Institute for Cell Therapy and Immunology, Leipzig, Germany

**Keywords:** SARS-CoV-2, Target identification, Target validation

## Abstract

The severe acute respiratory syndrome coronavirus 2 (SARS-CoV-2) is the causal agent of the COVID-19 pandemic. More than 274 million individuals have suffered from COVID-19 and over five million people have died from this disease so far. Therefore, there is an urgent need for therapeutic drugs. Repurposing FDA approved drugs should be favored since evaluation of safety and efficacy of de-novo drug design are both costly and time consuming. We report that imatinib, an Abl tyrosine kinase inhibitor, robustly decreases SARS-CoV-2 infection and uncover a mechanism of action. We show that imatinib inhibits the infection of SARS-CoV-2 and its surrogate lentivector pseudotype. In latter, imatinib inhibited both routes of viral entry, endocytosis and membrane-fusion. We utilized a system to quantify in real-time cell–cell membrane fusion mediated by the SARS-CoV-2 surface protein, Spike, and its receptor, hACE2, to demonstrate that imatinib inhibits this process in an Abl1 and Abl2 independent manner. Furthermore, cellular thermal shift assay revealed a direct imatinib-Spike interaction that affects Spike susceptibility to trypsin digest. Collectively, our data suggest that imatinib inhibits Spike mediated viral entry by an off-target mechanism. These findings mark imatinib as a promising therapeutic drug in inhibiting the early steps of SARS-CoV-2 infection.

## Introduction

In December 2019, a new respiratory disease appeared in Wuhan, China, and became a global pandemic within a few months^1–3^. The identified agent, a coronavirus, showed substantial similarity to a previous virus that lead to an outbreak in 2003 with the severe acute respiratory syndrome coronavirus (SARS-CoV-1), and were therefore named SARS-CoV-2^4^. The respective disease was named coronavirus disease 2019 (COVID-19) and has a mortality of around 2%, causing more than 5.3 million global death so far^5,6^. Since available medical interventions directly against COVID-19 are lacking and the pandemic is still ongoing, it is crucial to identify possible drug targets for preventing severe outcomes of infected individuals. Already FDA-approved drugs should be herby in the main focus of researchers considering long-lasting clinical trials for developing new medical intervention.

SARS-CoV-2 is a member of the *Coronaviridae* family that includes some highly pathogenic strains like middle east respiratory syndrome coronavirus (MERS-CoV) and SARS-CoV-1^7–9^. Members of *Coronaviridae* infiltrate the respiratory tract, SARS-CoV-2 by using angiotensin converting enzyme 2 (hACE2) as the entry receptor and transmembrane serine protease 2 (TMPRSS2) for priming^10,11^. After binding of SARS-CoV-2 surface protein (Spike) to hACE2, the virion undergoes membrane fusion or receptor-mediated endocytosis dependent on the presence or absence of TMPRSS2, respectively^9,12–14^. An initial step within the infection cycle is the consecutive cleavage of Spike at two sites S1/S2 and S2’ (S2) by furin, trypsin, endosomal cathepsin L and/or membrane protease TMPRSS2. The cleavage exposes the fusogenic peptide, causing the conformational change of Spike and promoting the fusion of virus and host membrane^7,10,11,15^.

In tissue culture, the Abelson tyrosine-kinase inhibitor, imatinib, inhibits SARS-CoV-1 and MERS-CoV infection, however the underlying mechanisms remain elusive^16–18^. Recent studies suggest that this is the case for SARS-CoV-2, as well^19–21^. Imatinib is an FDA-approved drug currently used for treating chronic myeloid leukemia (CML) and gastrointestinal stromal tumors (GIST). The therapeutic effect of imatinib in cancer derives mainly from inhibiting Abelson kinase 1 (Abl1) and Abelson-related gene protein (Abl2, also known as Arg)^22–24^. Both proteins are non-receptor tyrosine kinases with diverged cellular functions^25,26^.

It has been reported that imatinib blocks the early stages of SARS-CoV-1 and MERS infection by inhibiting fusion of the virions at the endosomal membrane^17^. Furthermore, based on the prevention of virus-induced syncytia formation, it has been argued that imatinib and other Abl-kinase inhibitors inhibit cell–cell fusion^18^. Although Abl2 depletion reduces SARS-CoV-2 infection, it is unclear whether Abl2 regulates both endocytic and cell membrane fusion^17^. We report here that imatinib inhibits SARS-CoV-2 infection and Spike-mediated cell–cell membrane fusion in Abl-kinases knockout cells suggesting an imatinib off-target effect. Furthermore, we show that imatinib did not impair hACE2-Spike-interaction but rather stabilized Spike protein and changed its sensitivity to trypsin digestion, which suggests a direct interaction of imatinib with Spike.

## Results

### Imatinib impedes early steps of SARS-CoV-2 infection

**Figure 1:**
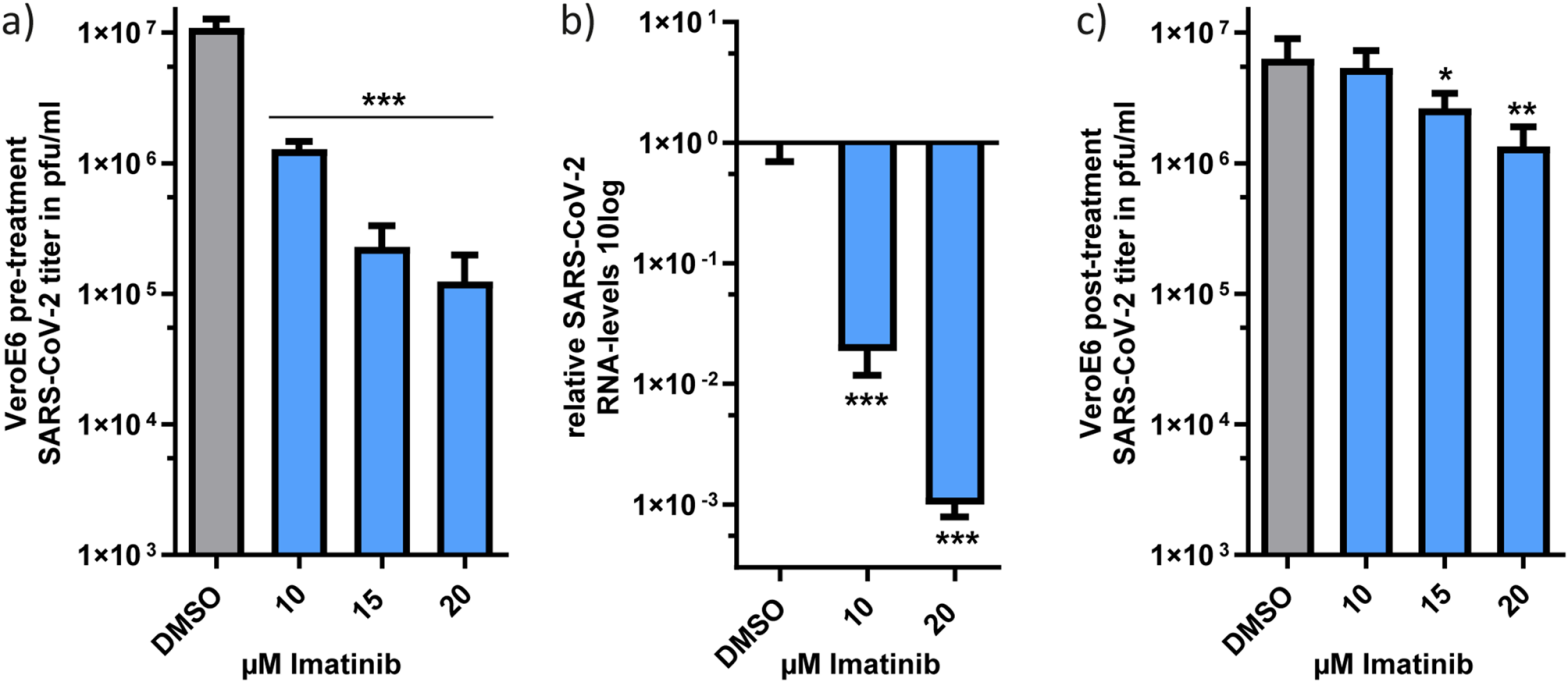
Imatinib diminished SARS-CoV-2 infection in VeroE6 cells. (**a**) Pre-treatment of imatinib reduced SARS-CoV-2 infection. Vero E6 cells were pretreated with increasing concentration of imatinib 2 h prior to infection with SARS-CoV-2 at 0.01 MOI, followed by 24 h incubation. The infectious virions were quantified by viral titration. Results represent one of three biological replicates. (**b**) Imatinib treatment strongly reduced SARS-CoV-2-RNA-levels within the infected cells. VeroE6 cells were imatinib treated and 2 h later infected with SARS-CoV-2. At 24 h, cells were harvested for RNA extraction. The level of viral RNA was quantified by real-time PCR and normalized to βActin RNA level. Results represent three technical replicates. (**c**) Imatinib treatment before SARS-CoV-2 infection is more effective than after infection. VeroE6 cells were infected with SARS-CoV-2 at 0.01 MOI and 2 h later treated with imatinib for 48 h. Results represent four technical replicates. In panels (**a**) to (**c**) data were statistically evaluated with One-Way ANOVA and Dunnett posttest. The data are normalized for the imatinib toxic effects according to XTT results of Fig. S1. *p ≤ 0.05; **p ≤ 0.01; ***p ≤ 0.001.

To investigate whether imatinib inhibits SARS-CoV-2 infection, we pre-treated VeroE6 with increasing concentrations of imatinib 2 h (h) before SARS-CoV-2 infection, and for additional 24 h, and then quantified SARS-CoV-2 viral titers in plaque-forming units per ml (pfu/ml)^27^. Imatinib treatment before infection significantly reduced infection by two orders of magnitude (Fig. 1a). Analysis of intracellular SARS-CoV-2 RNA revealed that viral RNA level was robustly reduced in an imatinib dose-dependent manner (Fig. 1b).

To pinpoint the time of maximal imatinib effect on infection, we also performed post-treatment analysis, by first infecting VeroE6, and then adding imatinib at 2 h post infection, for 48 h. Although a significant dose-dependent reduction of infection was observed (Fig. 1c), the level of inhibition was much lower at imatinib post-treatment, than that obtained with the addition of imatinib before infection (imatinib pre-treatment, Fig. 1a and c). These results suggest that imatinib inhibited the early steps of SARS-CoV-2 infection, although we cannot rule out the possibility that imatinib might inhibit late stages as well.

### Imatinib inhibits SARS-CoV-2 pseudovirus infection

**Figure 2:**
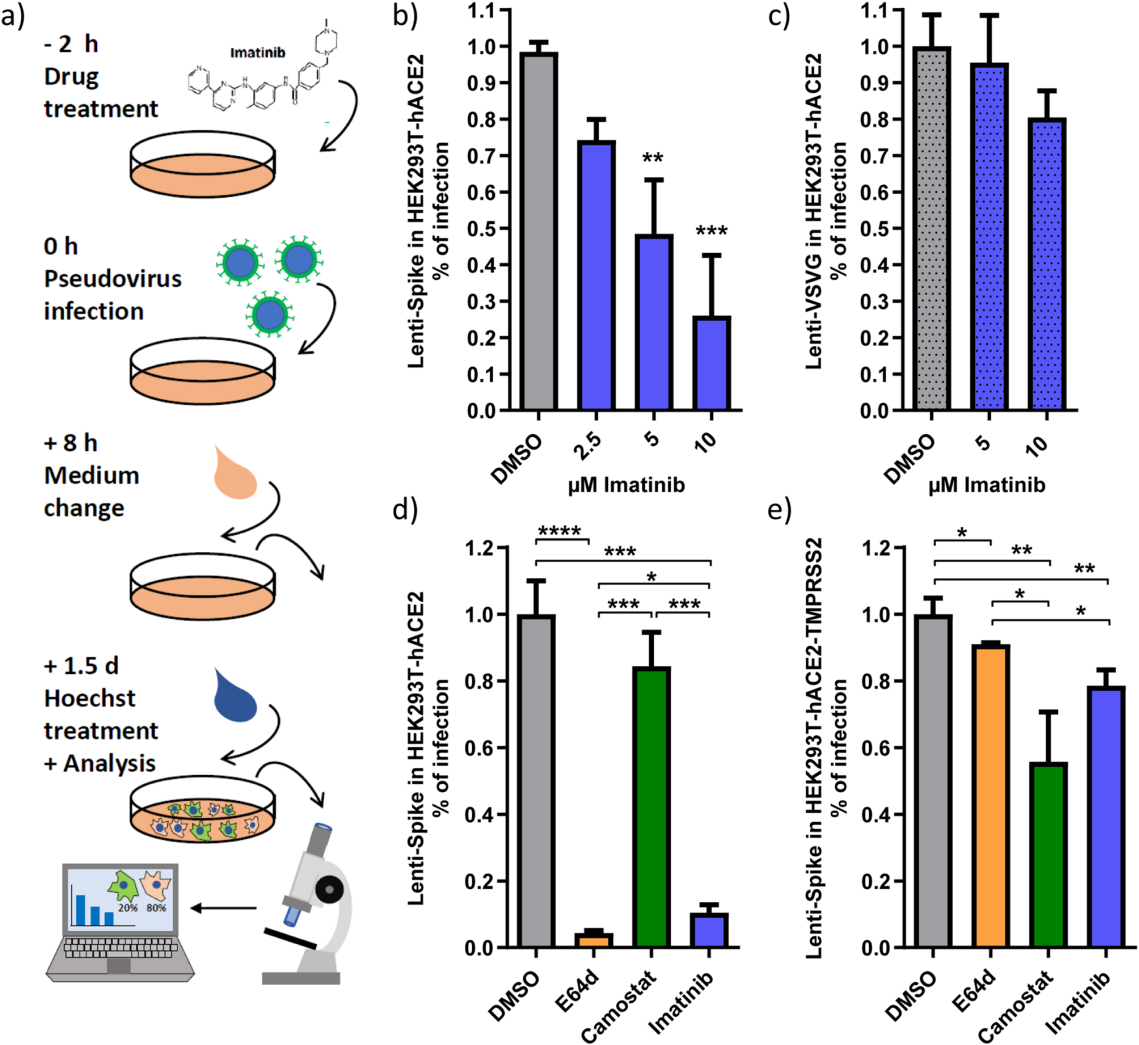
Imatinib specifically inhibited lenti-Spike infection in both TMPRSS2-negative and -positive cells. (**a**) A scheme of lenti-Spike experiments. HEK293T-hACE2 cells were treated with the respective drugs and 2 h later were infected on-top with lenti-Spike. Medium was replaced after 8 h with fresh growth medium. After 1.5 days, Hoechst was added to stain the cell nuclei. Ratio between total number of cells (Hoechst) and infected cells was calculated based on the transduced GFP signal. (**b**) Lenti-Spike infection is inhibited by imatinib. Cells were treated with different doses of imatinib 2 h prior to lenti-Spike infection. Infection-efficiency was determined 1.5 days later. (**c**) Imatinib inhibition is lenti-Spike specific. Cells were transduced with lenti-VSVG. (**d**) Imatinib inhibited lenti-Spike infection in TMPRSS2-negative cells. HEK293T-hACE2 were treated with each 25 µM E64d, 50 µM camostat or 10 µM imatinib, and infected with lenti-Spike 2 h later. (**e**) Imatinib reduced lenti-Spike infection in TMPRSS2-positive cells. HEK293T-hACE2 were transfected with pEFIRES-TMPRSS2 plasmid and puromycin was added a day later to select for HEK293T-hACE2-TMPRSS2 cells. Cells were treated the next day and infected as described. In panels (**b**) and (**c**) data were analyzed by One-Way ANOVA with Dunnett posttest. In panels (**d**) to (**e**) student-t-test was performed; *p ≤ 0.05; **p ≤ 0.01; ***p ≤ 0.001; ***p ≤ 0.0001.

Next, we generated lentivirus-based SARS-CoV-2 pseudoviruses and performed the experiments at biosafety level 2 conditions (BSL2). To this end, we truncated 19 amino acids from the C-terminus of the Spike protein in order to delete the ER localization signal and redirect it to the cell membrane^7,11^. The constructed lenti-SARS-CoV-2-Spike-pseudovirus (lenti-Spike) expressed GFP upon transduction, and infection efficiency was quantified by determining the ratio between infected and non-infected cells (Fig. 2a). We show that imatinib inhibited lenti-Spike infection in a dose-dependent manner (Fig. 2b). Strong inhibition of lenti-Spike infection was obtained when imatinib was added either 2 h prior to infection, at the time of infection, or mixed with lenti-Spike sample before transduction (Fig. S2a, b). Thus, we believe that optimal therapeutic application of imatinib would be obtained either at the first days of infection while virus is spreading within the body or as prophylactic use. Here too, a much lower level of inhibition was obtained when cells were treated 2 h after lenti-Spike infection (Fig. S2b). The inhibition is Spike specific since the infection of lentiviruses with vesicular stomatitis virus glycoprotein (VSV-G) was insignificantly affected by imatinib (Fig. 2c). Since no lenti-Spike replication is involved in these conditions, we concluded that imatinib acted at the level of lenti-Spike specific cell attachment or entry steps. Furthermore, HEK293T-hACE2 cells pretreated with imatinib for 2 h but washed-out shortly before lenti-Spike addition were well transduced (Fig. S2c), suggesting that imatinib is required at the time of infection.

### Imatinib inhibits both endocytosis and membrane fusion infection pathways

It was previously reported that the presence or absence of TMPRSS2 determines the SARS-CoV-2 entry route either via membrane fusion or endocytosis, respectively^10,14^. E64d, a cathepsin L inhibitor, significantly inhibits endosomal SARS-CoV-2 infection mainly in the absence of TMPRSS2 while camostat, a serine-protease-inhibitor, inhibits membrane-fusion-mediated infection in the TMPRSS2 positive cells^10^.

HEK293T do not express TMPRSS2 and cells ectopically expressing TMPRSS2 were more susceptible to infection with both SARS-CoV-2 and the related pseudovirions^10^. We observed similar behavior with lenti-Spike. In the absence of TMPRSS2, lenti-Spike infection of HEK293T-hACE2 was robustly inhibited with both E64d and imatinib but not camostat (Fig. 2d). However, in the TMPRSS2 positive cells, the level of inhibition by imatinib was significantly higher over that of E64d (Fig. 2e). While E64d and camostat effects are dependent on the absence or presence of TMPRSS2, respectively, imatinib was active in both cases, indicating that imatinib effectively inhibits both routes of infection; receptor-mediated-endocytosis and membrane fusion.

### Imatinib slows down Spike-mediated syncytia-formation

**Figure 3:**
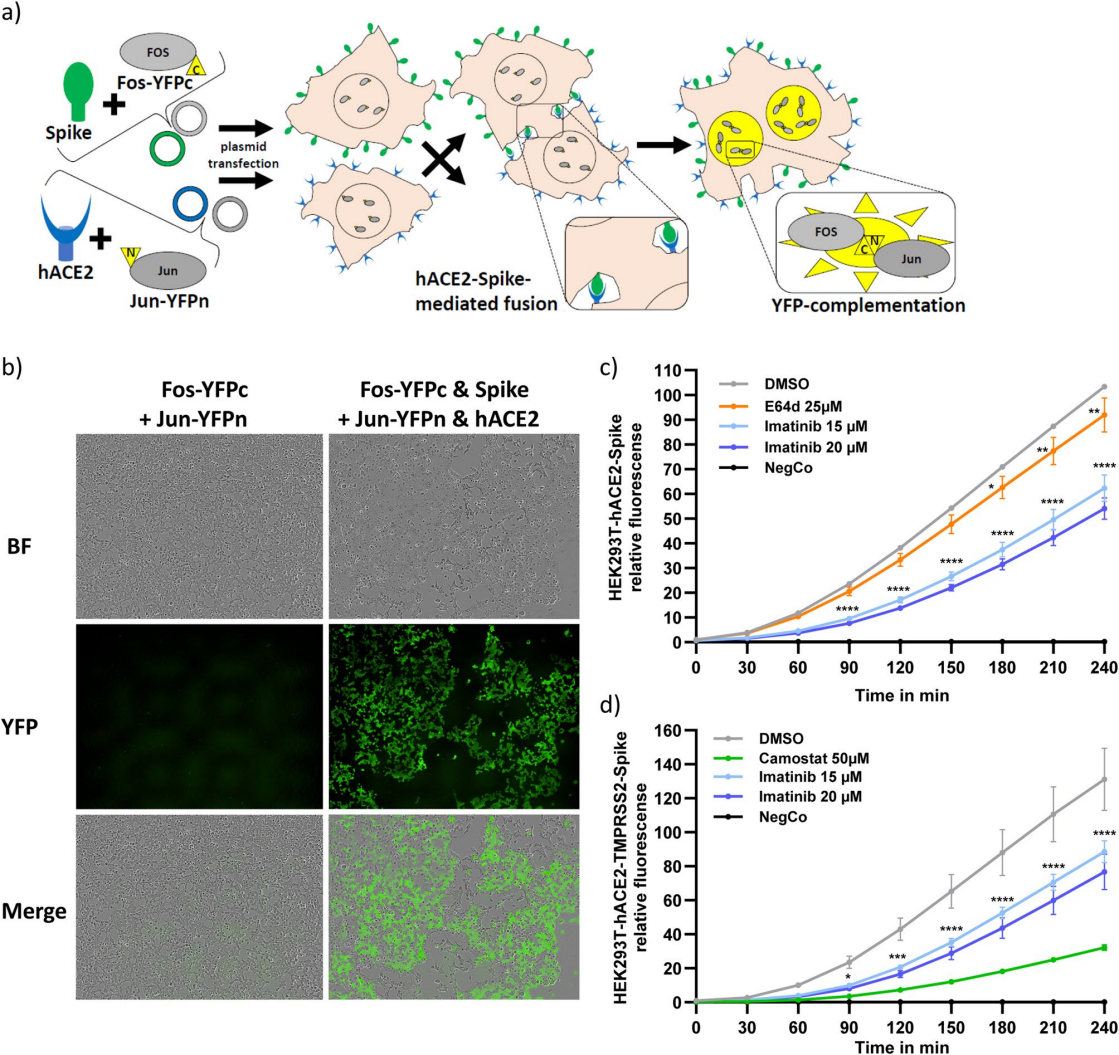
Imatinib delayed Spike mediated syncytia formation. (**a**) Scheme of cell-fusion-assay. HEK293T were transfected with plasmids containing either Fos-YFPc and Spike or Jun-YFPn and hACE2. The transcription factors Fos-YFPc and Jun-YFPn are located inside the nucleus; while hACE2 and Spike are localized at the cell-membrane. When cells mixed together, interaction of hACE2 and Spike lead to cell-fusion. Upon cell fusion Fos-YFPc and Jun-YFPn are heterodimerized and emit fluorescence signals. (**b**) HEK293T cell were transfected with either Fos-YFPc or Jun-YFPn in the presence or absence of hACE2 and Spike. After 2 days, cells were mixed and emitted YFP nuclear signal only in the presence of both hACE2 and Spike. Images represent fusion event 5 h after mixing of the cells. (**c**) Imatinib delayed Spike-hACE2 mediated fusion. HEK293T cells were transfected as done in panel (**b**) and 2 days later were harvested, counted, and treated with the indicated drugs for 2 h. Next, cells were mixed and the plates were immediately transferred in IncuCyte-Incubator. The fluorescens signal was monitored every 30 min and calculated by IncuCyte-software. (**d**) In TMPRSS2-positive cells, imatinib reduced the kinetic of syncytia formation. HEK293T were transfected with either mix of Fos-YFPc and Spike or Jun-YFPn, TMPRSS2 and hACE2. The data in panels (**b**) and (**c**) (N = 2) were analyzed with Two-Way ANOVA with Bonferroni multiple comparison and each represents one of three biological replicates: *p ≤ 0.05; **p ≤ 0.01; ***p ≤ 0.001; ****p ≤ 0.0001.

Syncytia formation, obtained by cell-to-cell fusion, characterizes *Coronaviridae* infection^13,28^. It describes a cytopathic effect promoting virion-free cell-to-cell spread, tissue damage and cell death^29^. Having demonstrated that imatinib inhibited the very early steps of infection, we next asked whether the inhibition is at the level of membrane fusion. To this end, we established a quantitative cell–cell membrane fusion assay. For visualizing and quantifying fusion events in real-time, we used a bimolecular fluorescence complementation (BiFC) approach. We transfected cells with either Spike and Fos-YFPc (C-terminal fragment of YFP) or hACE2 and Jun-YFPn (N-terminal fragment of YFP). Jun and Fos are nuclear transcription factors that heterodimerize in the nucleus. Cell nuclei become YFP positive only upon cell fusion and complementation of split-YFP via Jun-Fos heterodimerization (Fig. 3a, b)^30,31^. Cells were treated with imatinib 2 h prior to their mixing and YFP positive cells were quantified at different time points. Interestingly, imatinib significantly inhibited fusion kinetics dose-dependently, whereas E64d was barely active (Fig. 3c). Since E64d drug targets the endosome localized cathepsin L, it would explain why our assay of cell–cell fusion is E64d refractory^14,15,32^.

Next, we ectopically expressed TMPRSS2 to establish the HEK293T-hACE2-TMPRSS2 cell line. Imatinib inhibited HEK293T-hACE2-TMPRSS2 cell fusion kinetics as well (Fig. 3d). As expected, under these conditions, camostat robustly inhibited cell–cell fusion, validated the role of TMPRSS2 at the level of cell membrane fusion^14,15^. Imatinib slowed down Spike-mediated-fusion and the cytopathic syncytia formation in both TMPRSS2-negative and -positive cells. These results suggest that imatinib inhibited infection by diminishing the Spike-mediated fusion.

### Imatinib inhibits lenti-Spike infection in an Abl1 and Abl2 independent manner

**Figure 4:**
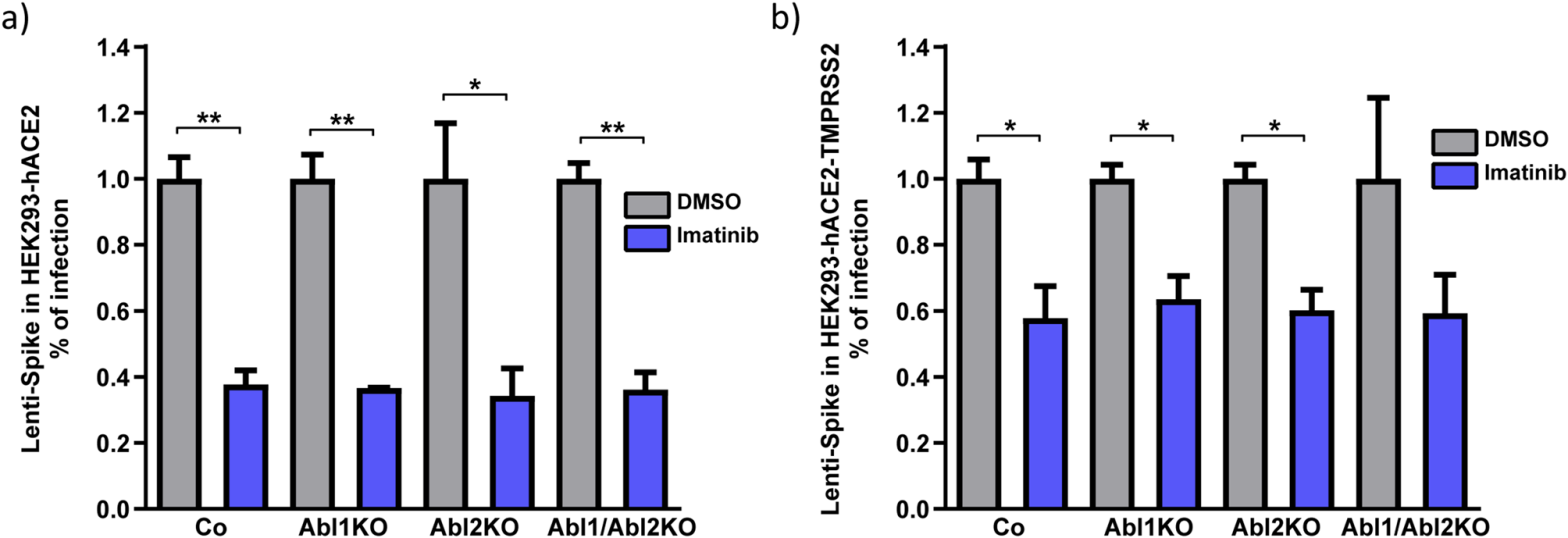
Lenti-Spike inhibiting effects of imatinib were Abelson-kinases independent. (**a**) Lenti-Spike inhibition by imatinib is Abelson-kinases independent in TMPRSS2-negative cells. HEK293-KO cells for each Abl1, Abl2 or both, were transfected with hACE2 2 days before infection. Lenti-Spike infection is reduced in the same level in all the tested cell lines when treated with 10 µM imatinib at the time of infection. Results represent means of two independent experiments with two biological replicates each. (**b**) Abelson-kinases are not responsible for lenti-Spike inhibition with imatinib in TMPRSS2-positive cells. The Abl-KO cell lines were transfected with TMPRSS2 and hACE2 2 days before lenti-Spike infection. Results represent means of two independent experiments with two biological replicates each and student-t-test was applied; *p ≤ 0.05; **p ≤ 0.01.

Next, we asked whether Abl1 or Abl2, the direct imatinib targets, are required for the early steps of pseudovirus infection. To this end, we created HEK293 Abl1 knockout (Abl1KO), Abl2KO and double Abl1/Abl2KO, two single clones of each (Figs. S3 and S4). We overexpressed hACE2 alone or together with TMPRSS2 in the KO cells and evaluated lenti-Spike infection and its susceptibility to imatinib. Differences at the level of transfection efficiency between Abl-KO-lines were evident, however, the normalized data revealed that imatinib inhibited lenti-Spike infection in all to the same level (Fig. 4a). This effect was also observed in the presence of TMPRSS2 (Fig. 4b).

Next, we monitored cell fusion rate in the established Abl-KO cell lines. A strong reduction at the level of cell–cell fusion was observed in imatinib-treated cells, in the presence and absence of TMPRSS2 (Figs. S5 and S6). These results suggest that imatinib inhibited lenti-Spike infection and Spike-mediated cell fusion in an Abl1 and Abl2 independent manner.

### Imatinib affects Spike conformation

**Figure 5:**
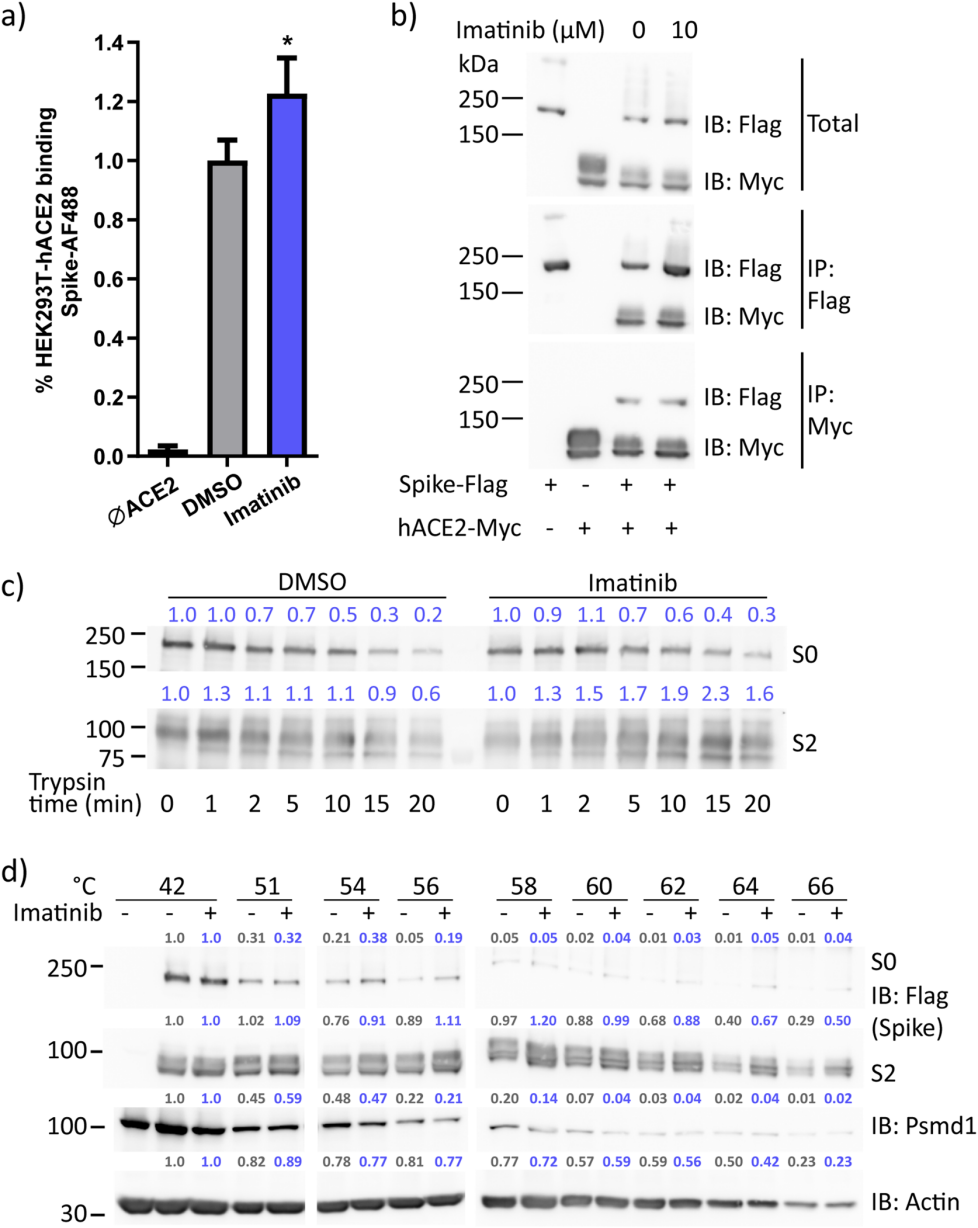
Imatinib affected Spike conformation via direct binding. (**a**) Imatinib increased the localization of Spike on cell surface. Spike-AF488 was added to HEK293T-hACE2 with or without 20 µM imatinib and incubated for 30 min on ice following 5 min in RT. The number of cells labeled with Spike-AF488 was quantified using flow cytometry. The data were analyzed by student t-test; *p ≤ 0.05. (**b**) Imatinib did not change Spike-hACE2 interaction. HEK293T were transfected with hACE2-Myc or Spike-Flag each alone or together and extracts were subjected to immunoprecipitation using the indicated antibodies. The immunoprecipitated proteins were analyzed by SDS-PAGE and immunoblotting (WB) using the indicated antibodies. Result represents one of two experiments. (**c**) Imatinib decreased the enzymatic cleavage rate of Spike. Spike-Flag was overexpressed, purified, and treated with either DMSO or 20 µM imatinib for an hour. Trypsin was added to a final concentration of 200 ng/ml and incubation was stopped at different time points by mixing the samples with Laemmli-buffer and boiling for 5 min. SDS-PAGE and WB was performed and position of S0 (unprocessed) and S2 (processed) Spike are indicated. Blue numbers indicate band intensity ratio compared to lane 0 min. Result represents one of three separate experiments. (**d**) Imatinib bound Spike, analysis by CETSA. Intact cells overexpressing Spike-Flag were treated with 20 µM imatinib or DMSO for 3 h, then incubated under increasing temperature (°C) and extracted. The insoluble fractions were removed by centrifugation and the soluble fractions subjected to SDS-PAGE and WB using the indicated antibodies. Actin and proteasomal subunit Psmd1 served as controls. Band intensity was calculated based on the values obtained at 42 °C. The numbers of DMSO and imatinib treatment are shown in gray and blue, respectively. Result represents one of three biological replicates.

Since imatinib reduced the rate of Spike-mediated infection and fusion in Abl independent manner, we next asked whether imatinib blocks hACE2-Spike-interaction. We performed a cell–protein-interaction-assay to quantify hACE2-Spike-binding at the cellular level. Fluorophore alexa-488 labeled Spike (Spike-AF488) was incubated with HEK293T-hACE2 cells and were either imatinib or DMSO treated on ice for 30 min. The number of cells labeled with Spike-AF488 was determined at room temperature using flow cytometry. No, reduction at the level of positive cells was evident in the imatinib-treated cells, but rather a slight increase, suggesting that imatinib did not inhibit hACE2-Spike-interaction (Fig. 5a).

A possible explanation for the extended localization of Spike after imatinib treatment could be either an increased Spike-hACE2 interaction or a reduced Spike cleavage rate. So, we employed the immunoprecipitation (IP) assay to quantify hACE2-Spike-interaction in the transfected cells. Flag-Spike IP brought down hACE2-Myc and the reciprocal experiment Flag-Spike was brought down by hACE2-Myc IP (Fig. 5b), validating their strong physical interaction. Here too, the interaction was imatinib refractory.

Next, we asked whether imatinib affects the cleavage rate of Spike. The cleavage of S0 to S2 exposes Spike fusion peptide, a critical step in virion-cell membrane fusion^7,15^. We isolated Flag-Spike from transfected cells and digested it in vitro with trypsin at different time points. We did not observe a change in the cleavage pattern, and the digestion of S0, the full-length Spike was only marginal protected by imatinib. The Spike cleavage product S2, however, was more trypsin resistant and accumulated in higher quantity in the presence of imatinib (Fig. 5c). These results suggest that imatinib influenced Spike conformation.

For validating imatinib-induced stabilization of Spike conformation, we used cellular thermal shift assay (CETSA)^33,34^, which allows studies of protein–ligand interactions in a cellular context. With increasing temperature, proteins are denatured, aggregated and insolubilized. This assay measures protein insolubility with increasing temperature which can change upon protein–drug interaction. Remarkably, we found that in the presence of imatinib the different Spike fragments are more stable at higher temperature (Figs. 5d and S7). These results further suggest that imatinib directly binds Spike and affects its conformation.

## Discussion

In this study, we provide evidence for imatinib inhibiting Spike mediated membrane fusion by an off-target mechanism. Infection of both SARS-CoV-2 and a surrogate lenti-Spike were blocked by imatinib (Figs. 1a, 2b), a widely used inhibitor of the non-receptor Abl1 and Abl2 tyrosine kinases. Given the fact that lenti-Spike is replication-incompetent, imatinib mode of action does not require viral replication. Further study revealed that imatinib inhibited membrane fusion mediated by Spike and hACE2 (Fig. 3c, d). In the absence of TMPRSS2, imatinib was as potent as the cathepsin L inhibitor (E64d) in blocking lenti-Spike infection (Fig. 2d), suggesting that imatinib inhibits infection in endosomal pathway. Interestingly, our results also show that imatinib is active in TMPRSS2 positive cells (Fig. 2e), where the lenti-Spike cell entry is mediated via virus-cell membrane fusion. This finding is further supported by quantifying cell–cell fusion. Under this experimental setting the infection’s endosomal route is eliminated and indeed the fusion is refractory to the cathepsin L inhibitor (Fig. 3c). However, in the presence of TMPRSS2, cell–cell fusion is blocked by TMPRSS2 inhibitor (camostat) and by imatinib (Fig. 3d). Since SARS-CoV-2 encompasses multi-organ tropism, it can infect various tissues, TMPRSS2-negative and -positive, as observed in severe COVID-19-cases^35–37^. The finding that imatinib inhibited both pathways of SARS-CoV-2 infection may point to imatinib’ superiority over other SARS-CoV-2 entry-inhibitors such as hydroxychloroquine (HCQ), E64d or camostat that are limited on their specificity to either endosomal or membrane fusion route of infection^10,14,15,38^.

Since imatinib is a relatively specific inhibitor of Abl1 and Abl2, the question is whether these tyrosine kinases participate in SARS-CoV-2 infection. In the context of SARS-CoV-1 and MERS, it has been reported that Abl2 knockdown reduces infection^17^. Since the employed cell lines are TMPRSS2 negative in the reported study, the involvement of Abl2 might likely be relevant to the endosomal route of infection^17^. The author’s conclusion that Abl-kinases participate in membrane-membrane fusion is based on quantification of virus-induced syncytia formation^18^. However, syncytia are formed upon Spike expression by the infected cells and therefore the observed reduction in the syncytia formation is the direct outcome of infection efficiency. Therefore, to investigate the role of Abl-kinases in membrane-membrane fusion more direct measurements are required. Here, we used protocols to selectively quantify infection via virus-membrane (Fig. 4b) and membrane-membrane fusion (Figs. S5 and S6) and found that the imatinib effect on these processes are both Abl1 and Abl2 refractory.

Computational studies predicted the direct binding of imatinib to hACE2 while others predicted its binding to the Spike^21,39–42^. We experimentally challenged the Spike binding prediction, both in a cell-free system and in cells and found that indeed imatinib binds Spike and changes its susceptibility to trypsin digestion (Fig. 5c) and stabilizes its conformation in high temperatures (Figs. 5d and S7), respectively. While computational studies predicted that imatinib reduces hACE2-Spike-interaction, we could not confirm this effect (Fig. 5a, b)^39–42^. Our findings demonstrate that imatinib binds Spike, introduces conformational changes and abrogates its processing rates. Given these findings and the finding that imatinib effects are Abl1 and -2 refractory, we concluded that imatinib is active by an off-target mechanism.

Interestingly, tyrosine-kinase-inhibitors show antiviral properties also against other viruses, including MERS, ebolavirus, coxsackievirus, hepatitis C virus (HCV), influenza and human immunodeficiency virus (HIV)^18,43–47^. Since these viruses do not share the same surface antigen, it is rather unlikely that imatinib has a similar off-target mechanism in these above-mentioned examples. In case of HIV, Abl-kinases influence wave2-signaling and in-turn hemifusion of virus and cell membrane^43^. In ebolavirus infection, Abl1 inhibits replication, but not entry^47^. Imatinib therefore can inhibit viral infection via both Abl kinases-dependent and -independent mechanisms.

Our results clearly demonstrate that imatinib is an effective SARS-CoV-2 entry-inhibitor. Therefore, imatinib therapeutic effects are optimally obtained upon its utilization at the prior or early stage of infection. But apart from Spike, additional imatinib-mediated mechanisms that impair SARS-CoV-2 infection may exist. For example, Yogalingam et al. showed that imatinib interferes with protein glycosylation possibly interfering with SARS-CoV-2 maturation since several viral proteins are known to be modified by carbohydrates^48–50^. Furthermore, imatinib treatment for 24 h significantly reduces ACE2 expression in both cell culture and mice. These effects were especially observed inside the lungs and could theoretically lead to less SARS-CoV-2-cell-interaction and consequently reduced infection^51^. Additionally, imatinib is known to suppress the immune system^52–54^. Since most SARS-CoV-2 patients die from an immunological overreaction known as cytokine storm, imatinib immunosuppressive effect could improve disease outcome of severe COVID-19 patients^55,56^. Therefore, immunosuppressants like dexamethasone are highly recommended for critically COVID-19 patients needing mechanical ventilation^57–59^. An initial clinical trial revealed that COVID-19 patients treated with imatinib show reduced mortality and shorter period of mechanically ventilation treatment compared with placebo group^60^. Indeed, the world health organization (WHO) regards imatinib as a promising drug in treating COVID-19^61^.

## Methods

### Cell culture

Human embryonic kidney cells over expressing SV40 large T-antigen (HEK293T, ATCC^®^), HEK293T expressing human angiotensin converting enzyme 2 (HEK293T-hACE2) or both ACE2 and TMPRSS2 (HEK293T-hACE2-TMPRSSS2) were cultured in Dulbecco’s modified eagle’s medium (DMEM, Gibco^®^) with 8% fetal bovine serum (FBS, Gibco^®^) and 100 units/ml penicillin and 100 µg/ml streptomycin (pen/strep; Biological Industries^®^). African green monkey kidney clone E6 cells (VeroE6, ATCC^®^ CRL-1586™) were grown in DMEM containing 10% FBS, MEM nonessential amino acids (NEAA), 2 mM L-glutamine, pen/strep and 12.5 Units/ml nystatin (P/S/N), all from Biological Industries^®^. All cells were cultured at 37 °C, 5% CO2 with 95% humidity. HEK293T-hACE2 cells were established by lenti-transduction of hACE2-4xMyc gene from pLenti6-hACE2-4xMyc construct and additional selection with 15 µg/ml blasticidin. In order to create HEK293T-hACE2-TMPRSS2, HEK293T-hACE2 were transfected with the respective plasmids 36 h before experimental setting and treated with 1 µg/ml puromycin 18 h before treatment. HEK293-Abl-KO cells derived from HEK293 TAF ts cell line as previously described^62^. KO was verified by Synthego ICE analysis for each clone (Figs. S3 and S4). For experiments, cells were non-enzymatically harvested with phosphate-buffered saline (PBS) + 1 mM EGTA. Between regular passages, cells detached from culture plate using trypsin solution B (Biological Industries^®^).

### Preparation of SARS-CoV-2 stock and infection

SARS-CoV-2 (GISAID accession EPI_ISL_406862) was kindly provided by Bundeswehr Institute of Microbiology, Munich, Germany. Virus stocks were propagated (four passages) and tittered on VeroE6. Handling and working with SARS-CoV-2 virus were conducted in a BSL3 facility in accordance with the biosafety guidelines of the Israel Institute for Biological Research.

VeroE6 cells were seeded in 24-well plates (2.5 × 10^5^ cells/well). Next morning, SARS-CoV-2 stocks were diluted in infection medium (MEM containing 2% FBS with NEAA, glutamine, and P/S/N) and used to infect VeroE6 in duplicates (200 µl/well) at 0.01 MOI. Imatinib pre-treatment experiments were performed by adding imatinib at respective concentrations for 2 h prior to SARS-CoV-2 infection. Following infection, cells were incubated for 24 h in MEM medium containing 2% FBS with NEAA, 2 mM L-glutamine, P/S/N and sodium bicarbonate (all Biological Industries^®^), and in the presence of imatinib at the appropriate concentration. At 24 h post infection, culture media (supernatant) were collected from each well for viral titration, while cell pellets were harvested for RNA. Imatinib post-treatment experiments were performed by adding imatinib at 2 h post SARS-CoV-2 infection, and incubation for 48 h. At 48 h post infection, supernatant was collected from each well for viral titration.

### Viral titration

For determination of viral titers of SARS-CoV-2 following pre- or post-infection imatinib treatment, VeroE6 were seeded in 12-well plates (5 × 10^5^ cells/well). 24 h later, supernatants from each of the above-mentioned treatment conditions were serially diluted in infection medium and used to infect VeroE6. Plates were incubated for 1 h at 37 °C to allow viral adsorption. Then, 2 ml/well of overlay (MEM containing 2% FBS and 0.4% tragacanth, Merck^®^) was added and plates were incubated for 72 h. The media were then aspirated, and the cells were fixed and stained with 1 ml/well of crystal violet solution (Biological Industries^®^). The number of plaques in each well was determined, and SARS-CoV-2 titers were calculated.

### RNA extraction and analysis

Cells were harvested and RNA isolated with TRI reagent (BioLab^®^) according to the manufacturer instructions. Reverse transcription was performed by using qScript cDNA synthesis kit (Quantabio) according to the manufacturer guideline. To measure viral SARS-CoV-2 RNA, we performed real time PCR using extracted cDNA, PerfeCTa SYBR^®^ Green FastMix^®^ and primer pair fw-GCCTCTTCTCGTTCCTCATCAC and rv-AGCAGCATCACCGCCATTG for SARS-CoV-2 and compared expression with housekeeping gene β-Actin and primer pair fw-ATCGTGCGTGACATTAAGGAG and rv-AGGAAGGAAGGCTGGAAGAG.

### Plasmids

pCMV3-SARS-CoV-2-spike plasmid (pCMV3-Spike) is a gift from Ron Diskin lab. Via PCR we truncated 19AA from C-terminal part or added Flag-tag at C-terminal part and subcloned it back in the same plasmid backbone (pCMV3-Δ19Spike or pCMV3-SARS-Spike-Flag). For the construction of pLenti6-hACE2-4xmyc we first cloned hACE2-4xmyc first into pENTR plasmid with restriction sites NcoI and KpnI. Afterwards we used restriction-free cloning method via Gateway^®^ LR-clonase-II (Invitrogen) and the manufactural protocol.

For pEFIRES-hACE2-4xMyc we used restriction sites NheI and MluI and for pEFIRES-TMPRSS2-Flag NheI and XbaI restriction sites. For cell-fusion-assay, pBiFC-VN173 (Addgene) was used to fuse Jun upstream to YFPn (Jun-YFPn), using HindIII and BglII restriction sites. pBiFC-CC155 (Addgene) was used to fuse Fos upstream to the YFPc (Fos-YFPc) using EcoRI and KpnI restriction sites.

### Transfection

All transfections were conducted using the calcium-phosphate (CaPO_4_) method, when cells reached 70–80% confluency. DNA mix was prepared containing 8 µg DNA and 1:10 of 2.5 M calcium chloride (CaCl_2_) in water. Next, same amount of 2X HEPES buffered saline (HBSx2) was dropwise added while vortexing. Finally, the obtained total mix was incubated for one min and dropwise added to cells. The medium was replaced with fresh growth medium after 8 h.

### Pseudovirus infection

In 6 cm plate, HEK293T were transfected with pCMV-Δ19Spike (1.5 µg), pGIPZ-tGFP (3.5 µg) and ΔR8.9 plasmids (3 µg). Medium was replaced with 3 ml growth medium after 8 h and cells were extracted 48 h later. The virus containing medium were filtered through 0.45 µm cellulose filter (Sartorius^®^) and used to transduce cells. Medium was changed after 8 h and analyzed after additional 24 h. Before analyzing, cell nuclei were stained with Hoechst solution. For estimating transduction efficiency, ratio of Hoechst-stained nuclei and GFP infected cells was calculated using the ImageJ software (macro see supplement).

### Cytotoxicity assay

VeroE6 or HEK293T-hACE2 cells were seeded 4000 or 8000 cells/well of 96-well plate. After 24 h growth medium was replaced with increasing concentration of Imatinib (Novartis^®^) and washed away after 8 h. Two days later cell viability was measured using the XTT cell proliferation kit (Biological Industries^®^). Cells were treated with camostat (Tocris^®^) and E64d (Caymanchem) in reported doses^10,15,63,64^.

### Immuno-precipitation (IP) and western blotting

Cells were harvested with cold PBS and centrifuged for 5 min at 3500 rpm at 4 °C. The obtained pellets were resuspended in RIPA-buffer containing protease inhibitor (APExBIO^®^). Then the cell lysates were centrifuged at 4 °C for 15 min, and the supernatant were mixed either with Laemmli buffer or for IP with Flag- or Myc- Beads (Sigma^®^) and incubated for 4 h at 4 °C in rotator. Before mixing IP fractions with Laemmli buffer, washing steps and elution with either Flag or Myc peptide (Sigma^®^) were performed. SDS-PAGE and immunoblotting were done using described protocol^65^. EZ-ECL kit (Biological Industries^®^) was used to enhance signal of horseradish peroxidase-conjugated secondary antibodies (Jackson Immuno Research Laboratories^®^). Recording was done with ImageQuant LAS 4000 (GE Healthcare^®^). The following primary antibodies were used in this study: mouse anti-Actin (Sigma^®^), mouse anti-HA (Sigma^®^), mouse anti-Flag (Sigma^®^), mouse anti-Myc (9E10; Weizmann Institute, Rehovot, Israel) and mouse anti-PSMD1 (Antibodyverify^®^).

### Cell-fusion-assay

Cells were transfected with either Spike and Fos-YFPc or hACE2 and Jun-YFPn and the control RFP-plasmid. After 1.5 days cells were non-enzymatically harvested and adjusted to a concentration of 1.0 × 10^6^ cells for 24-well-plate. Next, drugs were added to the cell suspension and incubated for 2 h with frequent gentle vortexing. Cells were divided in duplicates and mixed in 1:1 ratio and plated. Next, cells were transferred to IncuCyte system (Sartorius^®^), and pictures were taken in brightfield, GFP and RFP-channel in half an hour interval. Calculation of YFP intensity were done with IncuCyte intern software.

### Cell–protein-interaction-assay

Purified Spike binding fluorophore Alexa Fluor 488 (Spike-AF488) was added to HEK293T-hACE2 together with DMSO or 20 µM imatinib and low speed centrifuged for 7 min at 4 °C. Next, cells were incubated for 30 min on ice and gently harvested and washed in RT in buffer containing PBS with 1 mM EDTA and 2% FBS. The number of cells labeled with Spike-AF488 was quantified using flow cytometry.

### Cellular thermal-shift assay (CETSA)

CESTA was done based on the method of Jafari et al., 2014, with modifications for membrane proteins^34,66^. Cells were transfected with Spike-Flag, and 24 h following transfection cells were treated with 20 µM imatinib for 3 h. Cells were non-enzymatically harvested and prepared as suspensions in PBS supplemented with protease inhibitors, with or without 20 µM imatinib. Cells were aliquoted into a series of PCR tubes, which were then subjected to a three mins heat shock (42–66 °C) for generating melt curves, followed by three mins cooling at 25 °C, and snap frozen in liquid nitrogen. Samples were thaw, NP-40 was added (1% v/v final), and the cells were lysed by three freeze–thaw cycles in liquid nitrogen. Separation of remaining soluble proteins from aggregates was done by centrifugation at 17,000 g for 20 min at 4 °C. The supernatants were analyzed by SDS-PAGE followed by Western blot analysis which displays the levels of thermally stable proteins.

### Statistics and graphs

All statistical test and graphs were accomplished with the help of GraphPad Prism software. If not otherwise described all the experiments were conducted with three biological replicates. In most of the experiments each value is of three technical replicates. To perform statistical test, the standard deviation of the reference bar, set to 1.0, were calculated from all technical replicates. T-tests are two-tailed and statistical bars are standard deviations.

## Supplementary Information

Supplementary Information.
