# Supplementary material for: Imatinib inhibits SARS-CoV-2 infection by an off-target-mechanism
Source: Sci Rep. 2022 Apr 6;12:5758. doi: 10.1038/s41598-022-09664-1 (PMC8984672; doi:10.1038/s41598-022-09664-1)
Supplement: Supplementary file 1 — Supplementary Information. [file 41598_2022_9664_MOESM1_ESM.pdf]

Supplement:

Imatinib inhibits SARS-CoV-2 infection by an off-target-mechanism

Romano Strobelt[1], Julia Adler[1], Nir Paran[2], Yfat Yahalom-Ronen[2], Sharon Melamed[2], Boaz Politi[2], Ziv Shulman[3], Dominik Schmiedel[3, 4], Yosef Shaul*[1]

[1] Department of Molecular Genetics, Weizmann Institute of Science, Rehovot, Israel

[2] Department of Infectious Diseases, Israel Institute for Biological Research, Ness Ziona, Israel

[3] Department of Immunology, Weizmann Institute of Science, Rehovot, Israel

[4] current address: Fraunhofer Institute for Cell Therapy and Immunology, Leipzig, Germany

[*] Corresponding author, yosef.shaul@weizmann.ac.il

Supplementary figures

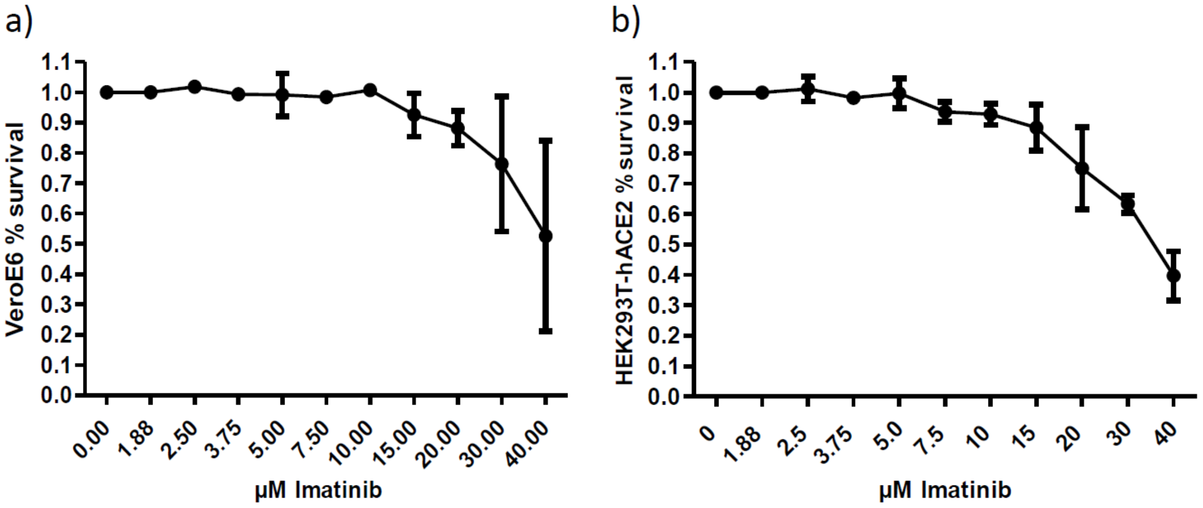

Figure S1: Cytotoxicity of imatinib in VeroE6 and HEK293T-hACE2 cells a) Imatinib is barley toxic to VeroE6 up to 20 µM. Cells were seeded in 96-well plate and next day treated with imatinib indicated doses for 8 hrs. After 2 additional days, cell survival was determined with XTT-assay. **b)** Imatinib is not toxic to the HEK293T cells up to 15 µM. HEK293T-hACE2 was treated with the indicated imatinib concentrations and cell survival was determined using the described protocol.

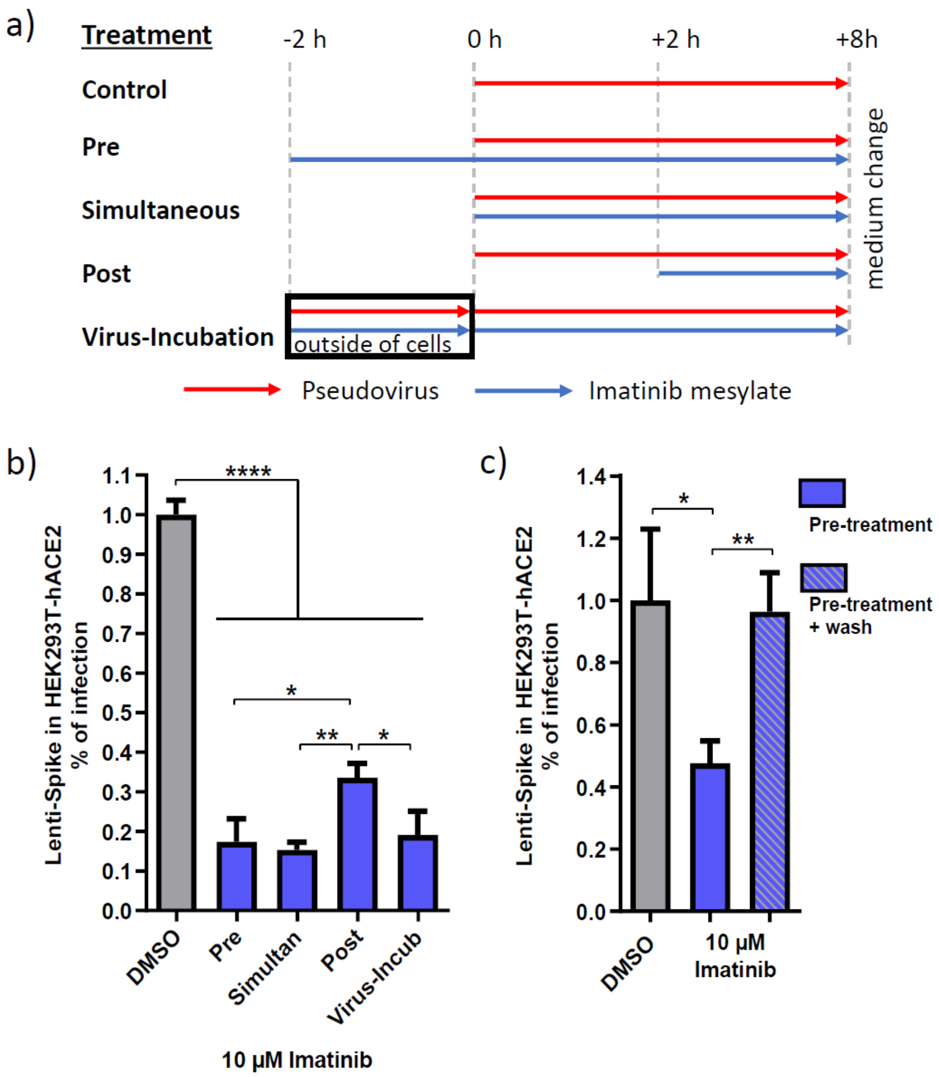

Figure S2: Maximal imatinib effect is obtained at the time of infection. a) Scheme of different imatinib treatment protocols for inhibiting lenti-Spike infection of HEK293T-hACE2. To design the best treatment protocol, imatinib was added to the cells either two hrs before (pre), simultaneous (simultan) or two hrs after (post) lenti-Spike infection. Another strategy was to incubate the virions with imatinib two hrs before adding the lenti-Spike to HEK293T-hACE2 cells (Virus-Incub). **b)** Imatinib strongly inhibited lenti-Spike infection in all the measured time points. Cells were treated with 10 µM imatinib with the indicated protocols and infection/treatment medium was replaced 8 hrs after Lenti-Spike infection. Hoechst was added after 1.5 days and infection efficiency was determined by calculating the ratio between infected and uninfected cells. **c)** Maximal imatinib inhibition is obtained at the time of infection. HEK293T-hACE2 cells were imatinib treated for two hrs and shortly before lenti-Spike infection, medium was replaced with either imatinib-free medium or imatinib-containing medium. Medium was changed after 8 hrs and infection efficiency was determined like above described. student-t-test; * = p≤0.05; ** = p≤0.01; *** = p≤0.001; **** = p≤0.0001

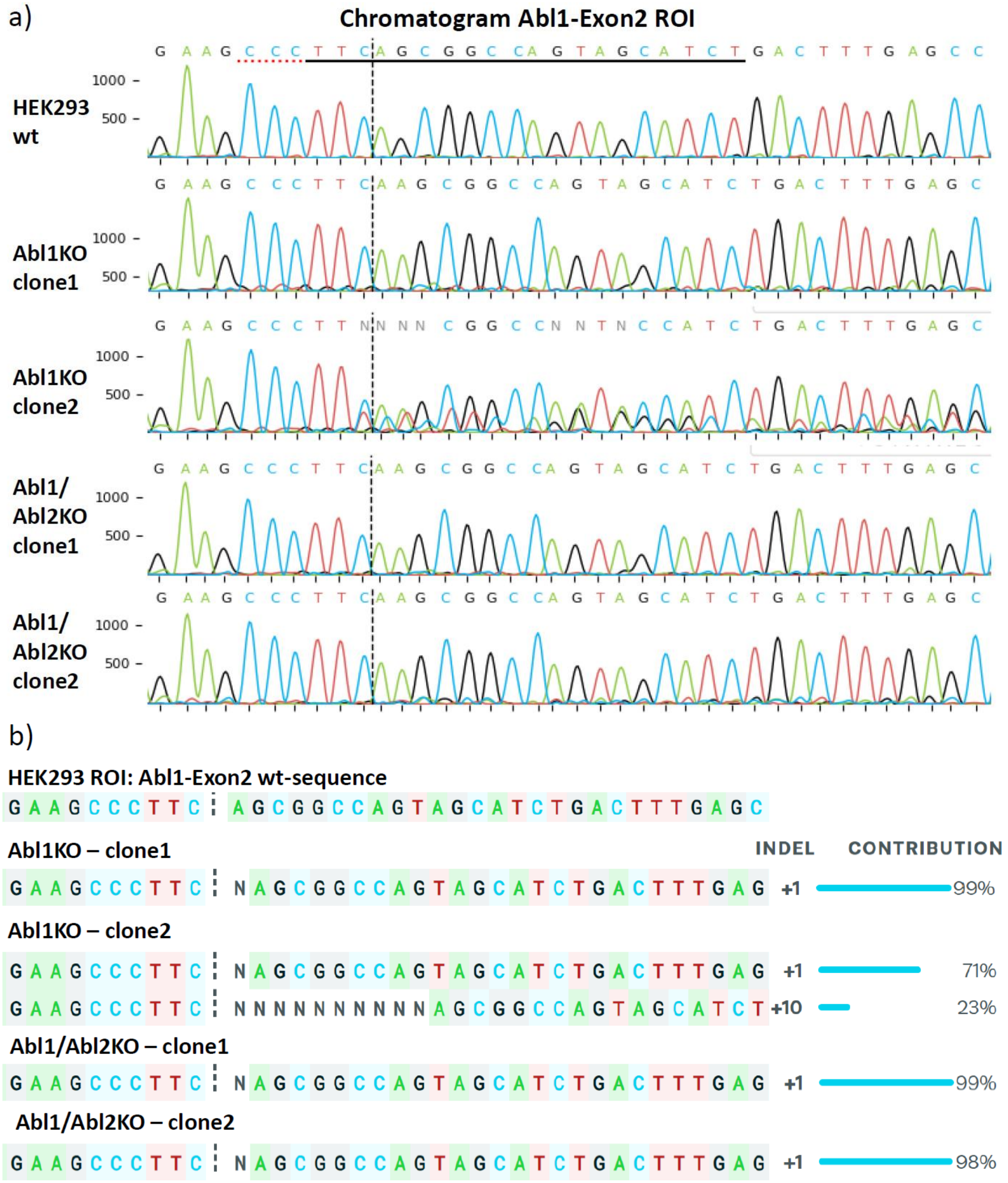

Figure S3: Sequence analysis of Abl1-KO a) HEK293 TAF ts cells were mutagenized at Abl1 exon2 using the published protocol[62]. Region of interest (ROI) in ABL1 gene was amplified using the primers fw-GGAACAATAATTACCACCTTGGAGTG and rv-GAACTCCTCTAAGGAGAGTCGACTCCC and sequenced. First chromatogram shows wt ROI sequence of HEK293 cells. The underlined sequence indicates Cas9-guide with relevant PAM sequence. Below the wt chromatogram the sequences of all the selected Abl1KO and Abl1/Abl2KO clones are shown. **b)** Chromatograms were evaluated with Synthego-ICE software tool. Both Abl1KO and the double Abl1/Abl2KO were devoid of wt sequence. All indels lead to frameshift mutations and premature stop-codons are expected within exon2. Since exon2 is included in all Abl1 isoforms, we assumed complete KO of Abl1 in the respective clones.

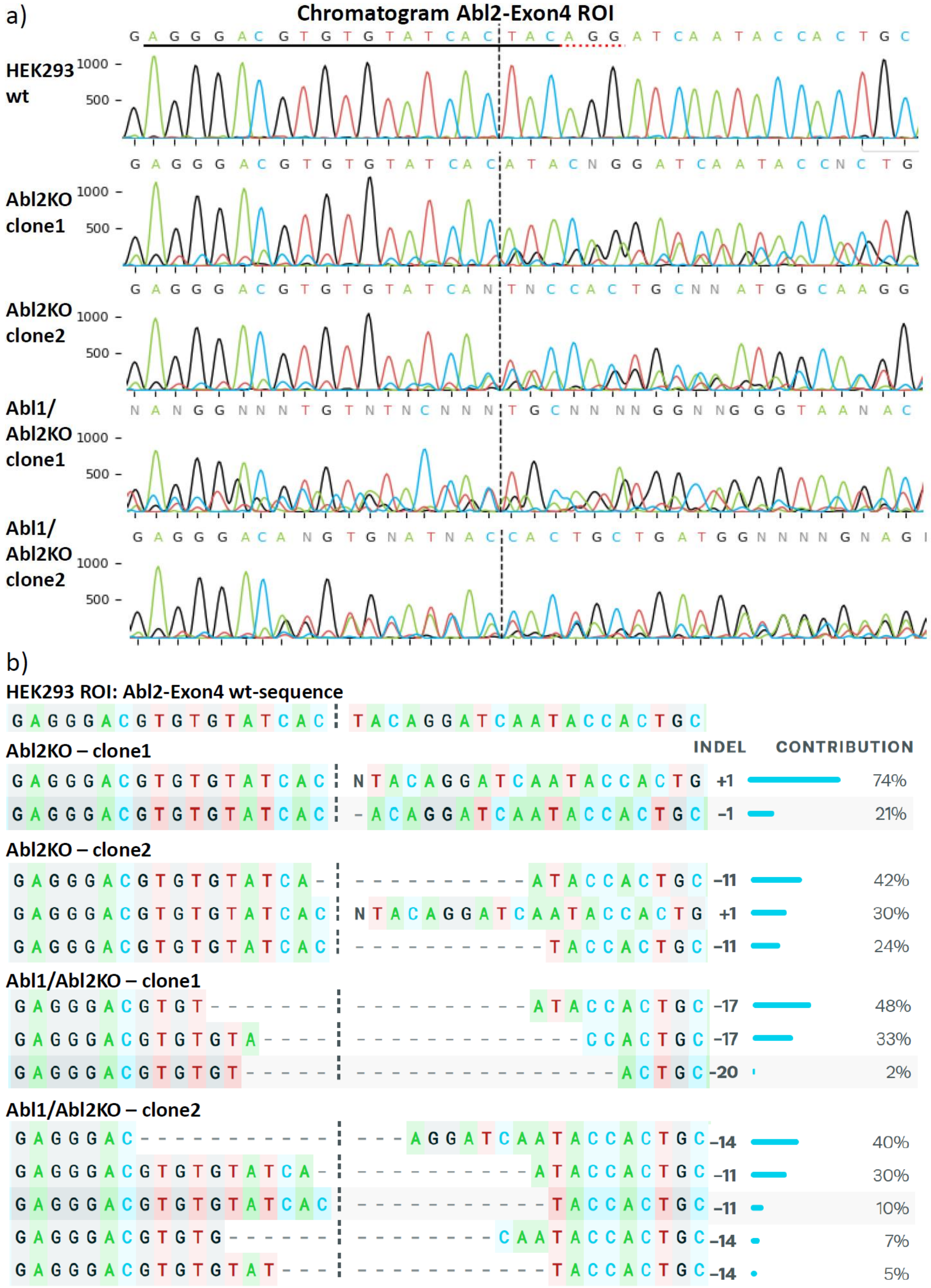

Figure S4: Analysis of HEK293KO clones for Abl2-KO a) HEK293 TAF ts were mutagenized at Abl2 exon4 using the published protocol[62]. The sequence of the Cas9-guide and the respective PAM is underlined in the first chromatogram, which shows the wt sequence of Abl2 gene exon4 ROI. ROI was amplified with the following primers; fw- AAAGCTACGAGTCCTTGGTTACAACCAGAA and rv- GTTCCCTTCATTCTTCACCTTCAAATCTAGC and sequenced. Chromatograms 2-4 show ROI of all the selected Abl2KO and double Abl1/Abl2KO clones. **b)** Abl2 ROI was evaluated with Synthego-ICE software tool. The selected KO clones bear indels but no wt sequence at Abl2 exon4. Furthermore, all indels result in frameshift mutations within Abl2 exon4 that would lead to premature stop-codons in exon5. Since exon4 and exon5 are included in all Abl2 isoforms, we can expect complete KO of Abl2 in the respective clones.

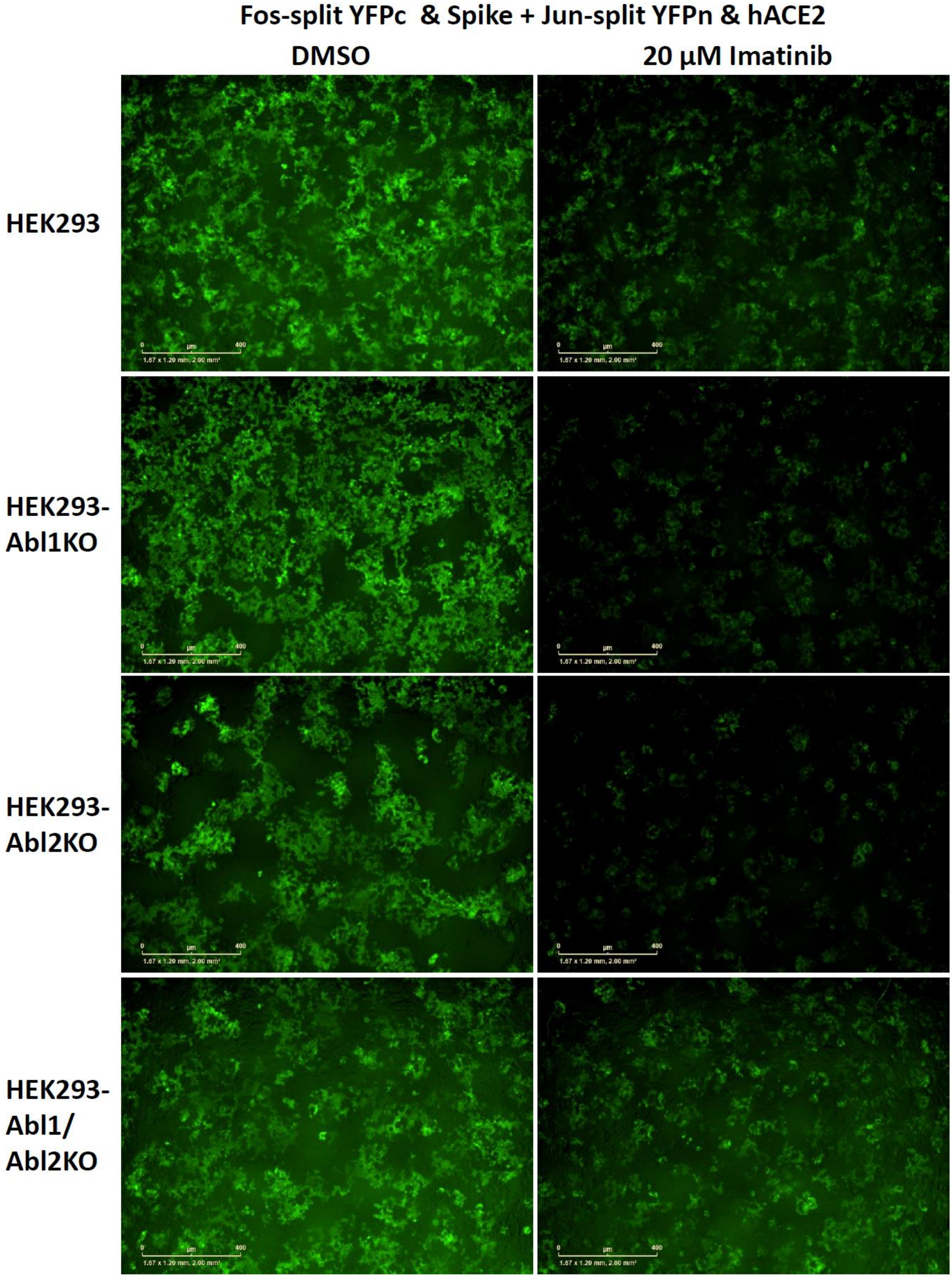

Figure S5: Imatinib inhibited Spike-mediated fusion in an Abelson-kinase independent manner. HEK293-KO cells were either transfected with Spike together with Fos-YFPc or hACE2 together with Jun-YFPn. After 1.5 days, cells were harvested, counted and treated with either DMSO or 20 µM imatinib. Two hrs later the two different generated cell lines were mixed in the 1:1 ratio in 96-well-plate and analyzed by IncuCyte-system. YFP-signal can be recorded only when the cells are fused and Fos and Jun are heterodimerized and YFP florescence is emitted. Pictures were taken every half an hour. Figures show cells 4 hrs after mixing and represent one of two biological replicates and one of three technical replicates.

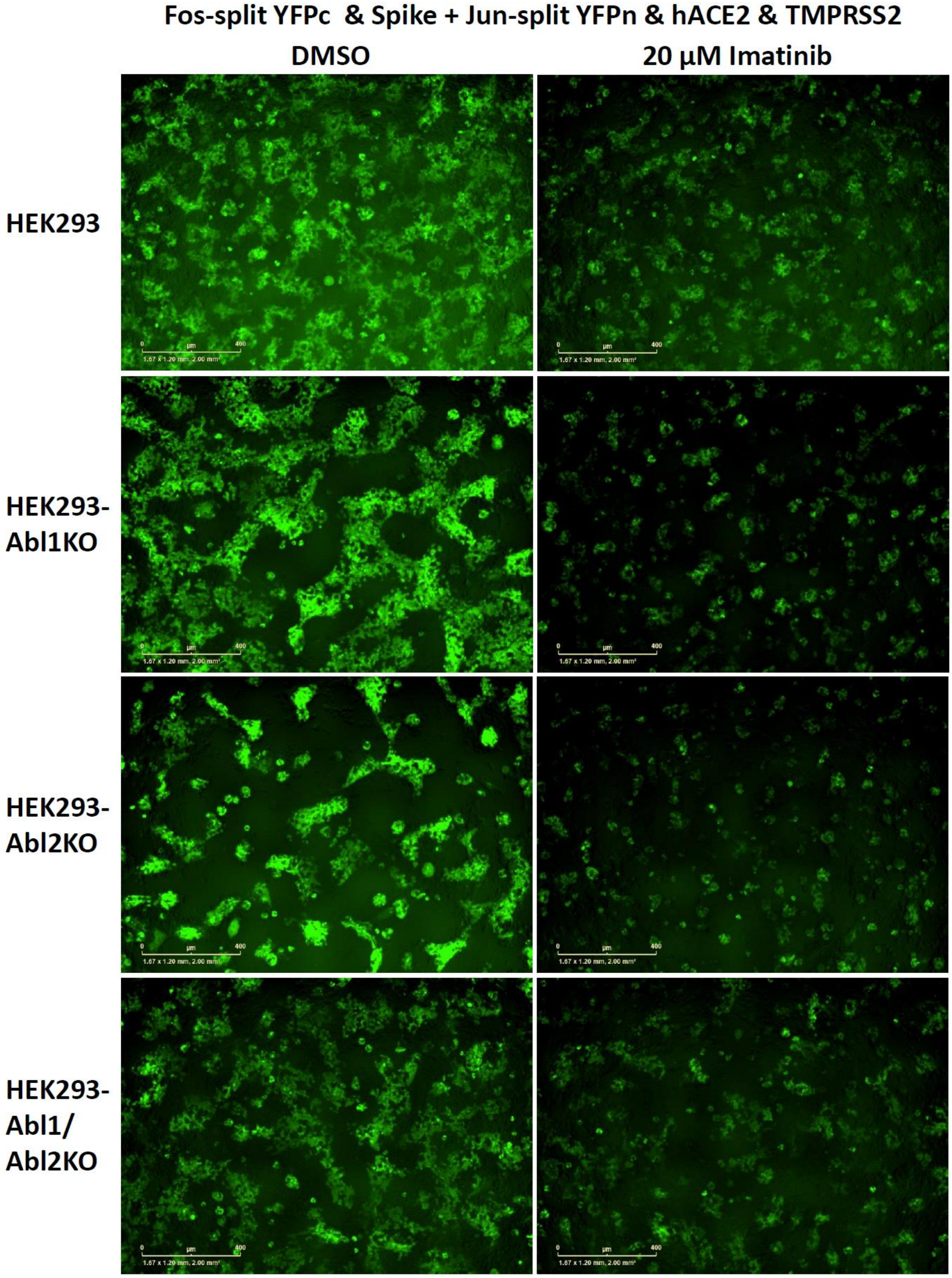

Figure S6: Spike-mediated cell fusion is inhibited by imatinib in TMPRSS2-positiv Abelson-kinase-KO cells. Imatinib is inhibiting Spike-mediated cell membrane fusion is Abelson-kinases independent. The HEK293-KO cells were either transfected with Spike together with Fos-YFPc or hACE2 together with both TMPRSS2 and Jun-YFPn. 1.5 days later, the two generated cell lines were treated with DMSO or 20 µM imatinib and were mixed in the 1:1 ratio 2 hrs later. IncuCyte-system was recording YFP-signal of fused cells in half an hour interval. Pictures were taken 4 hrs after mixing and represent one of two biological replicates and one of three different technical replicates.

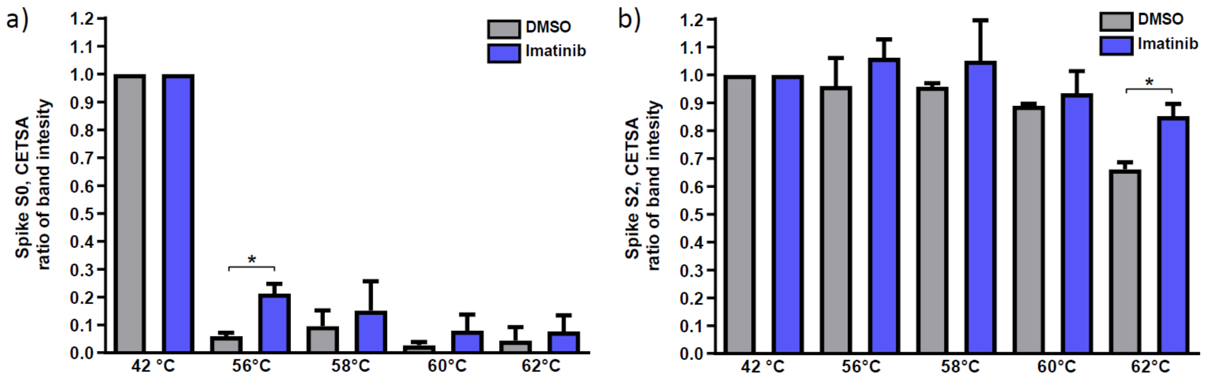

Figure S7: Measurements of CETSA experiments a) Analysis of Spike S0 band intensity of all three CETSA experiments. The 42°C DMSO and imatinib band intensity each was taken as 1.0 to calculate the ratio of higher temperature. **b)** Spike S2 band intensity of all three CETSA experiments. Values represents ratio to respective protein band at 42°C. student-t-test; * = p≤0.05

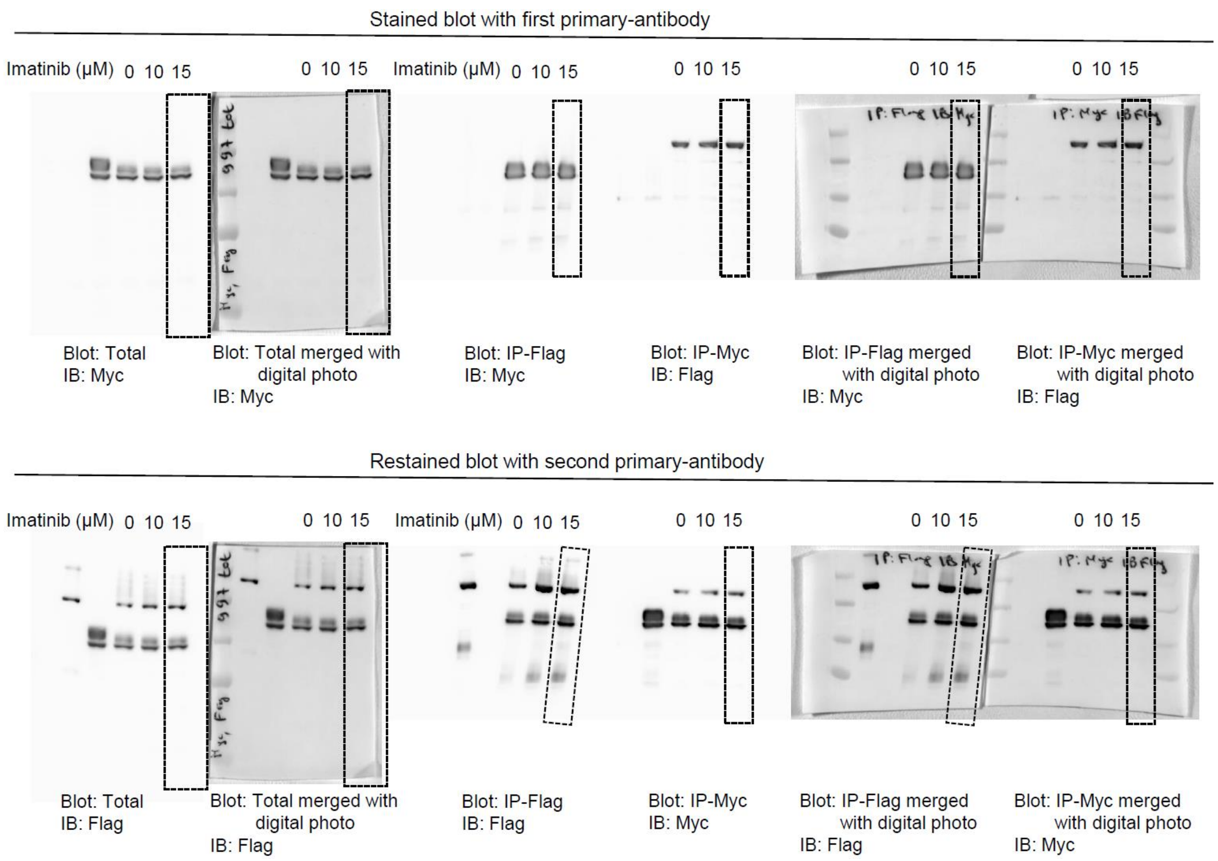

Figure S8: Original western blot image from co-IP figure 5b Dashed box shows excluded lanes from figure 5b in main text.

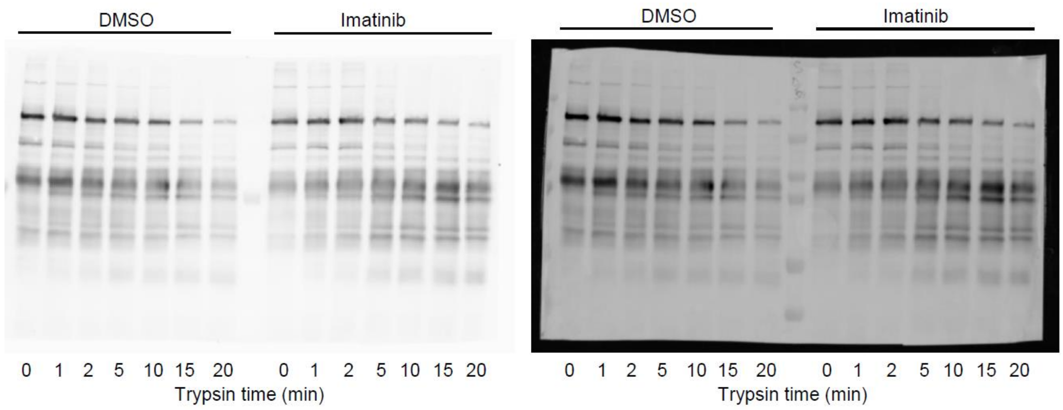

Figure S9: Original western blot image from trypsin digest figure 5c

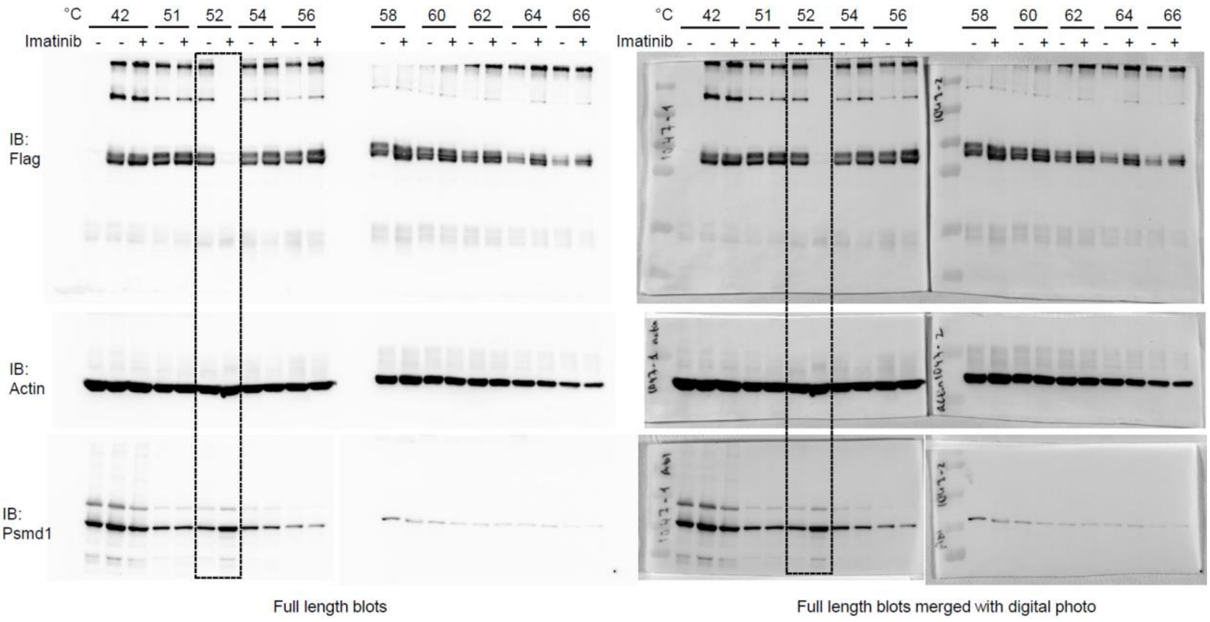

Figure S10: Original western blot image from CETSA figure 5d Dashed box shows excluded lanes from figure 5d in main text.

Supplement: ImageJ-Macro

Hoechst-Macro

Line 3 was adjusted for each experiment in respect to the intensity of fluorescence signal. Testing on several picture within each experiment ensured uniformity.

```
1. rename("Hoechst");
2. run("Duplicate...", " ");
3. setMinAndMax(0, 145);
4. run("8-bit");
5. run("Duplicate...", " ");
6. run("Gaussian Blur...", "sigma=3");
7. imageCalculator("Subtract create", "Hoechst-1","Hoechst-2");
8. selectWindow("Result of Hoechst-1");
9. run("Mean...", "radius=1.0");
10. setAutoThreshold("Default dark");
11. //run("Threshold...");
12. //setThreshold(7, 255);
13. setOption("BlackBackground", false);
14. run("Convert to Mask");
15. run("Dilate");
16. run("Fill Holes");
17. run("Dilate");
18. run("Watershed");
19. run("Analyze Particles...", "size=100-800 circularity=0.02-1.00 clear add");
20. selectWindow("Hoechst");
21. roiManager("Show All without labels");
22. roiManager("Measure");
23. run("Summarize");
```

GFP-Macro

Line 3 and 11 was adjust for each experiment in respect to the intensity of fluorescence signal. Testing on several picture within each experiment ensured uniformity.

```
1. rename("GFP");
2. run("Duplicate...", " ");
3. setMinAndMax(0, 120);
4. run("Apply LUT");
5. run("Subtract Background...", "rolling=60 sliding");
6. run("8-bit");
7. run("Duplicate...", " ");
8. run("Gaussian Blur...", "sigma=15");
9. imageCalculator("Subtract create", "GFP-1","GFP-2");
10. selectWindow("Result of GFP-1");
11. setMinAndMax(0, 10);
12. run("Apply LUT");
13. run("Mean...", "radius=1.0");
14. setAutoThreshold("Yen dark");
15. //run("Threshold...");
16. //setThreshold("Default dark", 0, 100);
17. setOption("BlackBackground", false);
18. run("Convert to Mask");
19. run("Dilate");
20. run("Fill Holes");
21. run("Dilate");
22. run("Watershed");
23. run("Analyze Particles...", "size=250-2000 circularity=0.01-1.00 clear add");
24. selectWindow("GFP");
25. roiManager("Show All without labels");
26. roiManager("Measure");
27. run("Summarize");
```
